# Correlation of Heart Rate Variability and Subjective Withdrawal Symptoms in Patients With Opioid Dependence, and Its Comparison in Patients Undergoing Detoxification With Patients Maintained on Opioid Agonist Treatment

**DOI:** 10.1192/bjo.2024.266

**Published:** 2024-08-01

**Authors:** Adit Verma, Siddharth Sarkar, Kanwal Preet Kochhar, Esha Sood, Dinu Chandran

**Affiliations:** 1 All India Institute of Medical Sciences, New Delhi, India; 2 Transitional Health Science and Technology Institute, NCR Biotech Cluster, Faridabad, India

## Abstract

**Aims:**

Patients with opioid dependence seek treatment for the discomforting withdrawal symptoms. Accurate clinical assessment is essential as medications are optimized based on these withdrawal symptoms. However, subjective reporting can present challenges. Heart rate variability (HRV) can enhance clinical assessment and has taxonomic and therapeutic implications. This study aimed to explore the correlation between HRV and subjective withdrawal in patients with opioid dependence and to compare the HRV parameters in patients undergoing detoxification to those maintained on opioid agonist treatment and healthy controls.

**Methods:**

3 groups of adult male participants were included. Group 1 included 40 patients with opioid dependence undergoing inpatient detoxification. Group 2 included 40 patients with opioid dependence receiving stable doses of buprenorphine on outpatient basis. Group 3 included 49 healthy controls. The Subjective Opiate Withdrawal Scale (SOWS) was used for withdrawal symptoms. For Group 1 and Group 2, HRV was assessed twice – before administration of morning dose of buprenorphine, and then 2 hours post administration. For Group 3 HRV was assessed once.

**Results:**

At baseline, resting heart rate differed significantly between the 3 groups (p < 0.001), it was highest for Group 2 (92.4) and lowest for Group 3 (79.4). In time domain parameters of HRV, the beat-to-beat variability was highest for Group 1 with standard deviation of all normal RR intervals (SDNN) = 134.8, root mean square of successive differences between normal heartbeats (RMSSD) = 181.7 and RR tri index = 8.9 (p < 0.005). In frequency domain parameters of HRV, total power was highest for Group 1 (98334.1, p < 0.001) while relative power did not differ significantly among the groups. The SOWS had a weak negative correlation with RMSSD in Group 2 (r = -0.312, p < 0.05) but did not have any correlation with HRV parameters in Group 1. Post administration of morning buprenorphine, the HRV parameters did not show a significant change in either of the groups (except reduction in very low frequency percentage in Group 1 from 12.013 to 7.196, p < 0.05).

**Conclusion:**

A higher degree of subjective withdrawal is associated with lower beat-to-beat variability in patients on stable doses of buprenorphine. However, this exploratory study did not find a robust relationship between HRV and subjective withdrawal symptoms. Higher RMSSD (representative of higher vagal tone) in patients undergoing detoxification may suggest greater physiological adaptation to withdrawal symptoms. This study provides additional insights into HRV in patients with opioid dependence.

